# Supplementary material for: Comparative Study of Sample Carriers for the Identification of Volatile Compounds in Biological Fluids Using Raman Spectroscopy
Source: Molecules. 2022 May 20;27(10):3279. doi: 10.3390/molecules27103279 (PMC9144713; doi:10.3390/molecules27103279)
Supplement: Supplementary file 1 [file molecules-27-03279-s001.zip › molecules-1663750-supplementary.pdf]

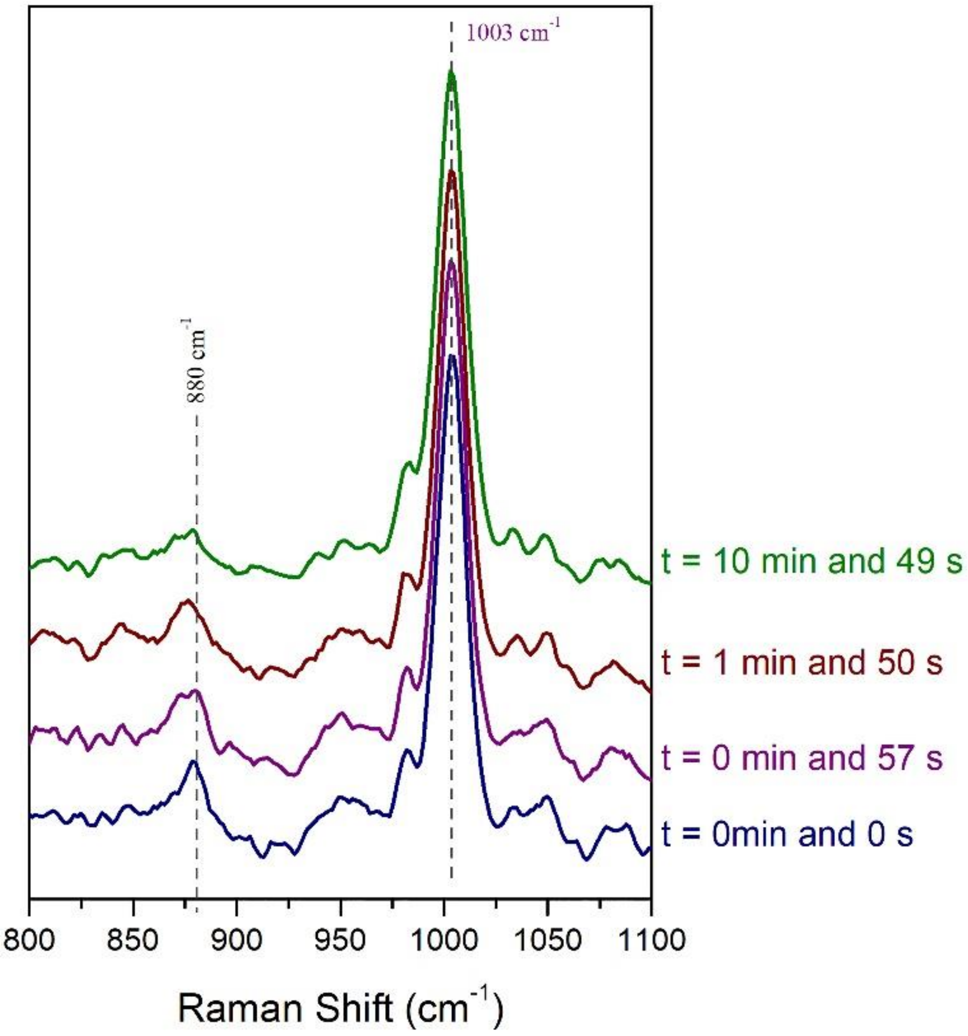

Figure S1. Raman spectra of the droplet of a 5 μL/mL ethanolic sample in human urine were acquired immediately (t = 0 min and 0 s) after droplet deposition on the gold coated glass slide, after 0 min and 57 s, 1 min and 50 s and 10 min and 49 s. The Raman spectra are normalized according to the urine peak at 1003 cm^{-1} and smoothed.

Table S1. The rate of change of the ethanol peak to urine peak intensities ratio with respect to time. The sample used consisted of 5 μL/mL ethanol in human urine, and the method used was the deposition of a droplet on gold coated glass slide.

Time	Rate of Change (%)
0 min and 0 s	0.00
0 min and 57s	-8.36
1 min and 50 s	-15.64
10 min and 49 s	-52.67

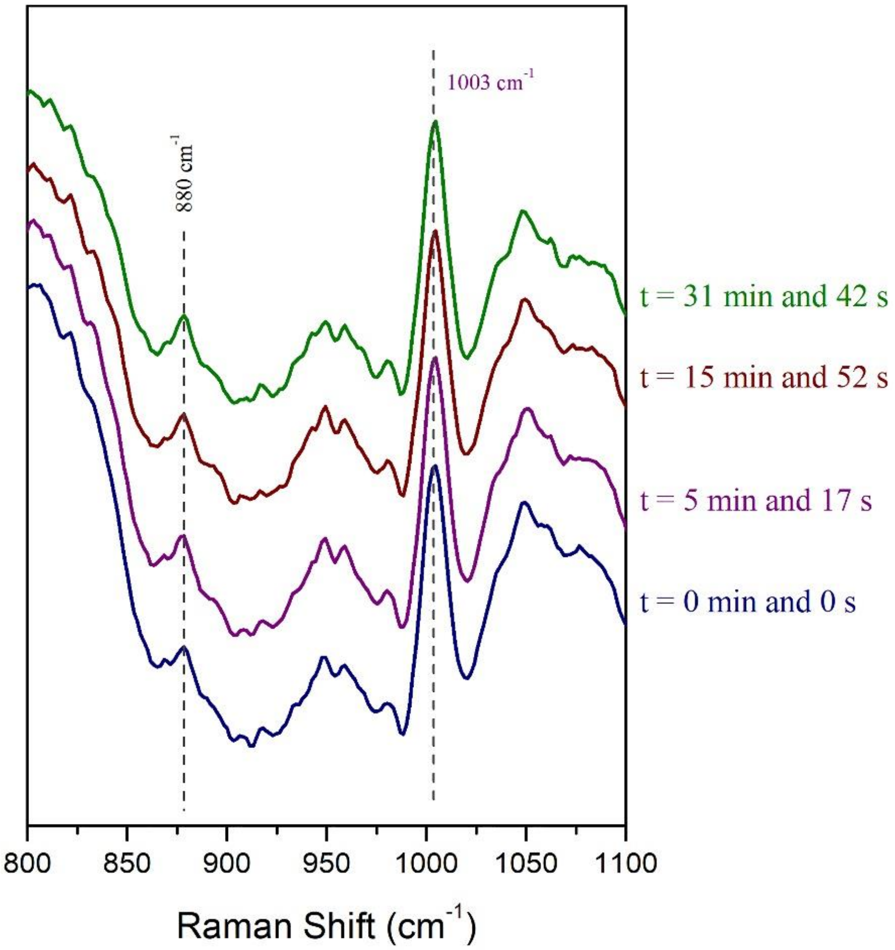

Figure S2. Raman spectra of a 5 μL/mL ethanolic sample in human urine were acquired immediately (t = 0 min and 0 s) after placing the sample in the commercially available cuvette for Raman spectroscopy with a mirror on the back side, after 5 min and 17 s, 15 min and 52 s and 31 min and 42 s. The Raman spectra are normalized according to the urine peak at 1003 cm^{-1} and smoothed.

Table S2. The rate of change of the ethanol peak to urine peak intensities ratio with respect to time. The sample used consisted of 5 μL/mL ethanol in human urine, and the method used was the addition of the sample in a commercially available cuvette.

Time	Rate of Change (%)
0 min and 0 s	0.00
5 min and 17 s	-1.35
15 min and 52 s	-5.42
31 min and 42 s	-12.28

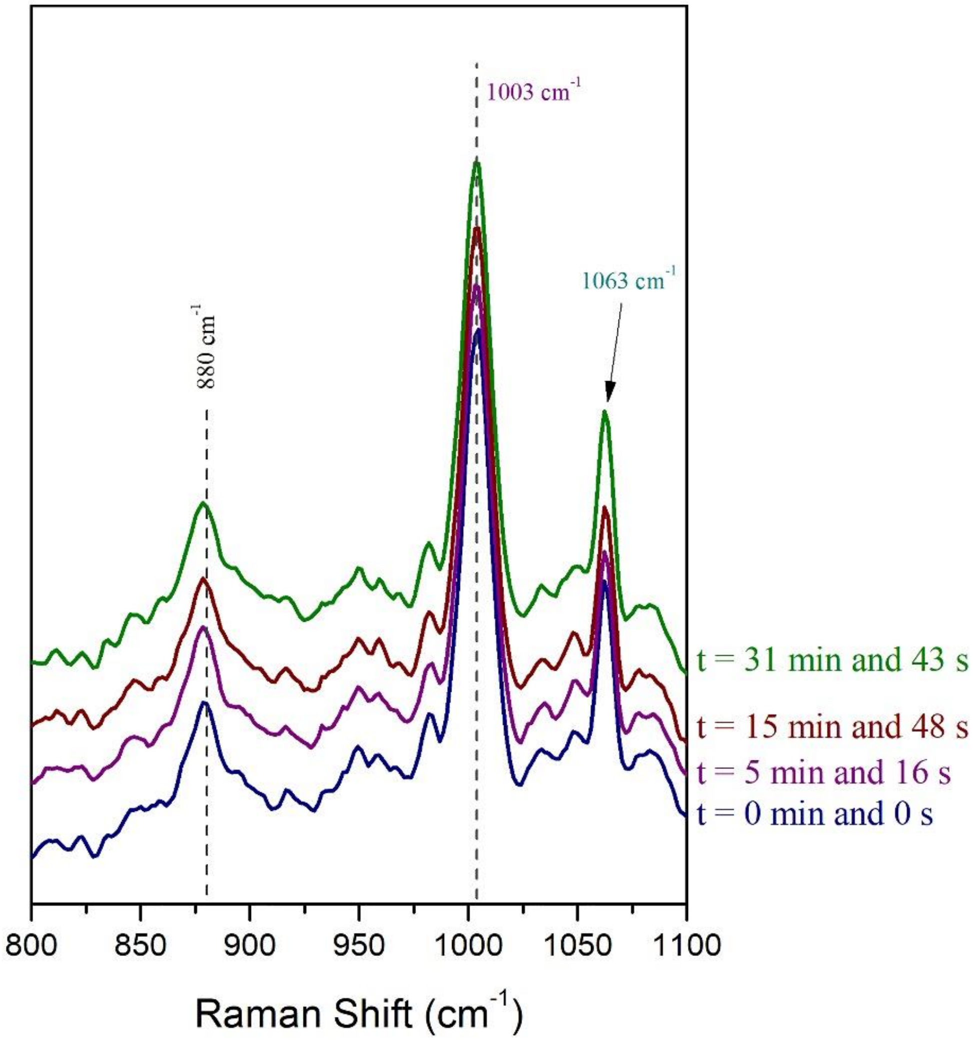

Figure S3. Raman spectra of 5 μL/mL ethanol in human urine were acquired immediately (t = 0 min and 0 s) after placing the sample in the cavity of the home designed glass microscope slide coated with a gold highly reflective substrate and covering the sample with a piece of transparent cling film (Vileda Freshmate®), after 5 min and 16 s, 15 min and 48 s and 31 min and 43 s. The Raman spectra are normalized according to the urine peak at 1003 cm^{-1} and smoothed.

Table S3. The rate of change of the ethanol peak to urine peak intensities ratio with respect to time. The sample used consisted of 5 μL/mL ethanol in human urine, and the method used was the deposition of the sample on gold coated glass slide with cavity, covered with transparent cling film.

Time	Rate of Change (%)
0 min and 0 s	0.00
5 min and 16 s	-8.19
15 min and 48 s	-15.44
31 min and 43 s	-20.83
